# Cancellous Bone May Have a Greater Adaptive Strain Threshold Than Cortical Bone

**DOI:** 10.1002/jbm4.10489

**Published:** 2021-03-30

**Authors:** Haisheng Yang, Whitney A Bullock, Alexandra Myhal, Philip DeShield, Daniel Duffy, Russell P Main

**Affiliations:** ^1^ Department of Biomedical Engineering, Faculty of Environment and Life Beijing University of Technology Beijing China; ^2^ School of Medicine Indiana University Bloomington IN USA; ^3^ Musculoskeletal Biology and Mechanics Lab, Department of Basic Medical Sciences Purdue University West Lafayette IN USA; ^4^ Weldon School of Biomedical Engineering Purdue University West Lafayette IN USA

**Keywords:** ADAPTIVE STRAIN THRESHOLD, BONE ADAPTATION, CANCELLOUS BONE, MECHANICAL LOADING, SOST/SCLEROSTIN

## Abstract

Strain magnitude has a controlling influence on bone adaptive response. However, questions remain as to how and if cancellous and cortical bone tissues respond differently to varied strain magnitudes, particularly at a molecular level. The goal of this study was to characterize the time‐dependent gene expression, bone formation, and structural response of the cancellous and cortical bone of female C57Bl/6 mice to mechanical loading by applying varying load levels (low: −3.5 N; medium: −5.2 N; high: −7 N) to the skeleton using a mouse tibia loading model. The loading experiment showed that cortical bone mass at the tibial midshaft was significantly enhanced following all load levels examined and bone formation activities were particularly elevated at the medium and high loads applied. In contrast, for the proximal metaphyseal cancellous bone, only the high load led to significant increases in bone mass and bone formation indices. Similarly, expression of genes associated with inhibition of bone formation (e.g., *Sost*) was altered in the diaphyseal cortical bone at all load levels, but in the metaphyseal cortico‐cancellous bone only by the high load. Finite element analysis determined that the peak tensile or compressive strains that were osteogenic for the proximal cancellous bone under the high load were significantly greater than those that were osteogenic for the midshaft cortical tissues under the low load. These results suggest that the magnitude of the strain stimulus regulating structural, cellular, and molecular responses of bone to loading may be greater for the cancellous tissues than for the cortical tissues. © 2021 The Authors. *JBMR Plus* published by Wiley Periodicals LLC on behalf of American Society for Bone and Mineral Research.

## Introduction

Mechanical loading is one of the critical factors that regulates and maintains skeletal health and bone mass. In humans, increased mechanical loading in the form of exercise can increase bone mass by modeling,^(^ ^1^, ^2^ ^)^ whereas reduced loading, such as spaceflight or long‐term bed rest, results in a loss of bone mass and skeletal integrity by remodeling.^(^ ^3^ ^)^ Osteocytes play a key role in directing bone (re)modeling through paracrine and autocrine signaling to modulate recruitment and lifespan of bone‐resorbing osteoclasts and bone‐forming osteoblasts.^(^ ^4^, ^5^, ^6^ ^)^

Mechanical loading‐induced bone tissue strain, or some factor related to it (e.g., strain rate), has been recognized as a driving force in the mechanical adaptation of bone and a signal that bone cells sense.^(^ ^7^, ^8^, ^9^ ^)^ In cortical bone, the concept of a minimum effective strain (MES) required to elicit an anabolic bone response can be combined with the number of applied load cycles to define a total strain stimulus that effectively predicts cortical bone adaptation in computational models.^(^ ^10^, ^11^, ^12^, ^13^, ^14^, ^15^ ^)^ *In vivo* loading experiments using the rodent axial ulnar loading or four‐point tibial‐bending models identified a MES value around 1000 με at the cortical sites examined in these studies.^(^ ^16^, ^17^, ^18^ ^)^ This MES value may be true near the mid‐diaphysis, but other cortical bone sites can exhibit different MES values, showing the variation possible in this value, depending upon bone site or loading protocol used.^(^ ^19^ ^)^ These *in vivo* loading models were limited to addressing only the cortical bone response to mechanical loading; thus, the MES for cancellous bone was not determined.

Skeletal sites rich in cancellous bone are more susceptible to osteoporotic fracture compared with diaphyseal cortical bone sites.^(^ ^20^ ^)^ Therefore, it remains an important goal to understand how cancellous bone is specifically regulated by strain magnitude. Similar to cortical bone, computational adaptive models are able to correctly predict the adaptation of cancellous bone density and alignment relative to the total strain stimulus.^(^ ^21^, ^22^, ^23^ ^)^ *In vivo* animal studies have been more equivocal. Based on a mouse caudal vertebral loading model, Webster and colleagues showed that the effect of loading on the structural and functional parameters of the cancellous bone is dose‐dependent.^(^ ^24^ ^)^ However, using a rabbit femoral cancellous loading model, Yang and colleagues found that trabecular bone adaptation to loading is not magnitude‐dependent at the strain levels examined.^(^ ^25^ ^)^ The axial tibial loading model can provide a more physiological mechanical environment to examine the effects of loading in both cortical and cancellous bone simultaneously.^(^ ^26^ ^)^ Similar to the response observed in other cortical loading models, cortical bone in the tibial loading model is dose‐dependent and governed by a load threshold.^(^ ^27^, ^28^, ^29^, ^30^, ^31^ ^)^ Available evidence suggests that the anabolic load threshold is greater in cancellous bone compared with cortical bone.^(^ ^29^, ^31^ ^)^ In these studies, the strains induced in the cortical and cancellous tissues were incompletely known because cancellous strain is impossible to measure directly *in vivo* and was not modeled in these studies. Given the expected importance of tissue strain in regulating bone formation response to load and the strain heterogeneity present in whole‐bone loading models,^(^ ^26^, ^32^ ^)^ this is an important shortcoming in our understanding for how cortical and cancellous bone respond to specific applied strain levels.

Even less well known are how gene regulation of bone formation and resorption are altered by varied strain magnitudes, the timescale for these changes, and if these signals differ between cortical and cancellous tissues. Several studies have shown time‐dependent gene expression profiles for cortical bone in response to mechanical loading.^(^ ^33^, ^34^, ^35^, ^36^, ^37^ ^)^ Reduced mechanical load magnitude (through disuse) has shown some evidence of downregulation of bone formation pathways.^(^ ^33^, ^34^ ^)^ Recently, transcriptional profiling of cortical *versus* cancellous bone from loaded mouse tibiae revealed differential time‐dependent gene expression patterns.^(^ ^38^ ^)^ Understanding the gene‐level response to increased mechanical stimuli could be critical in determining the basis for the variety of structural responses described for cortico‐cancellous bone tissues and potentially the underlying control mechanisms involved in bone mechanobiology.

The goal of this study was to examine the time‐specific gene expression, bone formation, and the volumetric structural response of cortical and cancellous bone to mechanical loading by applying varying load levels to the skeleton using axial tibial loading. We hypothesized that the cancellous bone response to loading will occur at a greater load threshold than for mid‐diaphyseal cortical bone, as has been shown by some previous studies.^(^ ^29^ ^)^ We also expect that relative gene expression associated with bone formation and resorption in the cancellous bone may be governed by a greater load threshold than cortical bone. However, for a given load, cancellous strains will be lower than mid‐diaphyseal cortical strains.^(^ ^32^, ^39^ ^)^ Thus, it remains unknown whether the strains required to induce molecular, cellular, and structural responses in cancellous bone will be greater than those described for cortical bone.

## Materials and Methods

### Animals

Female C57Bl/6 mice were obtained at 15 weeks of age (Jackson Laboratory) and housed five per cage with *ad libitum* access to water and standard rodent chow diet. The mice used in this study (*N* = 181) were divided between the experimental loading procedures as outlined in Fig. S1. All procedures were approved by the Purdue Animal Care and Use Committee (#1110000008).

### Relationship between applied load and induced strain in the tibia

In a separate group of mice, at 16 weeks of age, a single element strain gauge was attached to the surface of the medial mid‐diaphysis of the tibia in each mouse (n = 7), one leg at a time, following previously described methods.^(^ ^32^, ^40^ ^)^ The results of these strain gauge‐based measures have been published previously for these mice as part of a rigorous finite element sensitivity analysis for the mouse axial tibial loading model.^(^ ^32^ ^)^

### Characterization of cortical and cancellous strains by μCT‐based finite element analysis

The computational procedures and analyses to determine the relationship between applied load and induced strain environment throughout the cortical and cancellous bone in the tibiae of these same mice have been previously described.^(^ ^32^, ^39^, ^41^ ^)^ Briefly, the tibiae from the 16 wk‐old female mice that were used to determine tibial stiffness prior to the loading experiments were scanned by μCT at an isotropic voxel size of 10 μm. The μCT images of each tibia were input into a MATLAB‐based mesh‐generation and processing program to produce a three‐dimensional finite element (FE) mesh model consisting of tetrahedral elements. Heterogeneous material properties were assigned to the FE model based upon the grayscale values in the μCT scans. The loading and boundary conditions were applied to the tibia to replicate the *in vivo* compressive‐loading configuration. Comprehensive sensitivity analyses were performed to determine the optimized model parameters to match the predicted strains with gauge‐measured strain at the medial surface of the tibial midshaft.^(^ ^32^ ^)^

The maximum (tensile) and minimum (compressive) principal strains, as well as the absolute principal strains (abs) induced by the three load magnitudes examined here, were characterized across the metaphyseal cancellous (10% bone's length) and midshaft cortical (2.5% bone's length) volumes of interest (VOIs; Supplementary Information Fig. S1). The principal strain (abs) for any element is the largest absolute principal tensile or compressive strain calculated for that element. The cortical and cancellous VOIs are analogous to the μCT and histomorphometry VOIs described below. Consistent with our previous studies,^(^ ^32^, ^39^, ^41^ ^)^ the cut‐off values for the upper 95th percentile of the maximum or minimum principal strains in each VOI were defined to represent the peak tensile or compressive strains engendered in each VOI during axial compression loading, respectively. Defining the peak tensile or compressive strains using this method eliminates any anomalous high‐strain elements, which could be associated with unexpected numerical errors that can arise during FE modeling.

### In vivo tibial loading experiments

At 16 weeks of age, mice received unilateral *in vivo* dynamic compressive loading of the tibia (n = 174). Following Protocol III presented in Main and colleagues,^(^ ^26^ ^)^ peak dynamic compressive loads of −3.5 N (low), −5.2 N (medium), and − 7.0 N (high) were applied to the left hindlimb with a single load session consisting of 216 total load events (Supplementary Information Fig. S1 *A*,*B*). Right tibiae were used as nonloaded contralateral controls. Based upon the load‐strain relationship determined above by strain‐gauging and finite element analysis (FEA), these loads corresponded to induced periosteal surface strains of +694 με, +1032 με, or + 1389 με at the gauge site on the medial mid‐diaphysis (Table 1).

**Table 1:** The Peak and Mean Compressive and Tensile Strains in the Midshaft Cortical, Proximal Metaphyseal Cancellous, and Proximal Cortico‐Cancellous Volumes of Interest (VOIs), as well as the Measured Strains at the Gauge Site on the Medial Surface of the Mouse Tibiae Under Axial Compressive Loads of −3.5 N, −5.2 N, and − 7 N

VOIs	Low (−3.5 N)		Medium (−5.2 N)		High (−7 N)	
	Peak comp (mean comp)	Peak tens (mean tens)	Peak comp (mean comp)	Peak tens (mean tens)	Peak comp (mean comp)	Peak tens (mean tens)
Proximal cancellous	−1322 ± 160 ^a^ (−496 ± 54)^a^	852 ± 120 ^a^ (337 ± 40)^a^	−1964 ± 237 ^a,b^ (−737 ± 80)^a^ ^,^ ^b^	1266 ± 178 ^a^ (501 ± 60)^a^	−2644 ± 319 ^a,b^ (−993 ± 108)^a^ ^,^ ^b^	1705 ± 240 ^a,b^ (675 ± 80)^a^ ^,^ ^b^
Proximal cortico‐cancellous	−1150 ± 125 ^a^ (−495 ± 54)^a^	948 ± 149 ^a^ (398 ± 52)^a^	−1708 ± 186 ^a,b^ (−735 ± 80)^a^ ^,^ ^b^	1408 ± 221 ^a^ (592 ± 78)^a^ ^,^ ^b^	−2299 ± 250 ^a,b^ (−989 ± 108)^a^ ^,^ ^b^	1896 ± 298 ^a,b^ (796 ± 105)^a^ ^,^ ^b^
Midshaft cortical	−1469 ± 104 (−639 ± 49)	1294 ± 117 (504 ± 44)	−2183 ± 154 ^b^ (−949 ± 73)^b^	1923 ± 173 ^b^ (749 ± 65)^b^	−2939 ± 208 ^b^ (−1277 ± 99)^b^	2588 ± 233 ^b^ (1008 ± 88)^b^
Strain at gauge site	694 ± 105		1032 ± 156		1389 ± 210	

*Note*: Data are presented as mean ± SD values for seven mice. The peak compressive or tensile strains are the 95th percentile minimum or maximum principal strains, which indicate the cutoff strain values that include elements within the top 5% for each VOI.

Abbreviations: comp, compressive; tens, tensile.

^a^ *p* < 0.05 vs respective midshaft cortical bone, by paired *t* test.

^b^ *p* < 0.05 vs respective midshaft cortical bone of the low load (−3.5 N), by paired *t* test.

Mice were subjected to dynamic cyclic loading for a single loading session (3h– and 24h–load groups; n = 12/load group), three loading sessions over 3 days (3d load group; n = 12/load group), or 10 load sessions over 2wks (5 days/week; 2wk–load group; n = 22/load group; Supplementary Information Fig. S1 *C*). Mice were euthanized and the tibiae collected 3h or 24h after a single load session (3h, 24h), 24 h following three daily load sessions (3d), or 72h following 2wks of loading (2wk; Supplementary Information Fig. S1 *C*). The shorter‐term gene‐level responses in the tibiae to applied load were analyzed in the 3h, 24h, and 3d groups by qPCR (n = 12/load group). The long‐term gene‐ and cell‐level and structural response of the tibiae to 2wks of applied load were examined by qPCR (n = 12/load group), dynamic histomorphometry, and μCT (n = 10/load group) in the 2wk group (Fig. S1 *D*). All mice were euthanized by cervical dislocation, except for the mice loaded for 2wks that were used for histomorphometric and μCT analyses, which were euthanized by CO_2_ asphyxiation.

### μCT imaging and analysis

Whole tibiae (left and right) were dissected for the 2wk loading group (n = 10/group), fixed for 24h in 10% neutral buffered formalin, then stored in 70% ethanol. Tibiae were scanned by μCT at 10 μm (μCT40, Scanco). After determining bone length using whole‐bone scans, a volume extending 2.5% of the bone length, centered at the midshaft, was analyzed for cortical midshaft geometry, including cortical area (Ct.Ar; mm^2^), total area (Tt.Ar; mm^2^), medullary area (Ma.Ar; mm^2^), cortical thickness (Ct.Th; mm), and the maximum and minimum moments of inertia (Imax and Imin; mm^4^). A volume extending 10% of the bone's length was analyzed at the proximal tibial metaphysis, distal to the growth plate, for analysis of bone volume fraction (BV/TV; %), trabecular thickness (Tb.Th; mm), and trabecular separation (Tb.Sp; mm).^(^ ^42^ ^)^ Thresholds of 0.41 and 0.34 g HA/cm^3^ were used to segment the proximal cancellous and midshaft cortical bone, respectively, for all tibiae from the loading experiment. The thresholds were determined according to previous methods^(^ ^39^, ^40^ ^)^ and confirmed by visual inspection.

### Dynamic histomorphometry

In the mice subjected to 2 weeks of tibial loading (2wk), calcein fluorochrome injections (20 mg/kg, ip) were administered at 10 and 4 days prior to euthanasia (Supplementary Information Fig. S1 *C*). The tibiae obtained from a subset of mice (n = 4–6/load group), which were also used for μCT, were processed for histomorphometry. The bones were embedded in methyl methacrylate and thick sections (100 μm) cut at the tibial mid‐diaphyses using a diamond‐embedded wire saw (Histosaw; Delaware Diamond Knives) and ground to a final thickness of approximately 40 μm. For both the loaded and control limbs, single‐ and double‐labeled perimeter (sL.Pm, dL.Pm) and interlabel width (Ir.L.Wi) were measured on the periosteal (Ps.) and endocortical (Ec.) surfaces using a microscope equipped with an ultraviolet light source. Commercial software (Osteomeasure; OsteoMetrics) was used to measure mineralizing surface (MS/BS; %), mineral apposition rate (MAR; μm/day), and bone formation rate (BFR/BS; μm^3^/μm^2^/day). The terminology and units used are those recommended by the Histomorphometry Nomenclature Committee of the American Society for Bone and Mineral Research.^(^ ^43^ ^)^

Frontal‐plane thin sections (4 μm) from the loaded and control tibiae were cut by microtome (Reichert‐Jung 2050; Reichert‐Jung) and used to measure bone formation in the proximal metaphyseal cancellous bone. From mounted, unstained sections, cancellous MS/BS, MAR, and BFR were measured over an area of about 3 mm^2^, located distal to the primary spongiosa. A single section was analyzed per tibia for both mid‐diaphyseal cortical and metaphyseal cancellous analyses.

### Gene expression analyses in the loaded tibiae

The diaphyses and metaphyses of the loaded and control tibiae were analyzed for changes in the expression of genes related to bone formation and resorption (Supplementary Information Fig. S1 *D*). Following euthanasia, the tibiae (n = 12/experimental group) were cleaned of muscle and other soft tissues and the epiphysis removed just below the growth plate using a scalpel. The proximal metaphysis was defined by measuring 1.8 mm (10% bone length) from the proximal cut end, isolating this segment with a scalpel, and snap freezing in liquid nitrogen. The distal end of the tibia was removed just below the tibia–fibula junction and the cortical segment flushed with 1× PBS to remove the majority of the bone marrow cells (Supplementary Information Fig. S1 *D*). Flushed cortical segments were snap frozen in liquid nitrogen; all bone samples were stored at −80°C until further processing.

Left and right tibiae were stored and processed separately for the loaded groups. Bone samples were combined (n = 2 mice/sample for a final n = 6 samples/experimental group) and pulverized using ceramic mortar and pestle while frozen. Samples were then placed into 1‐ml Trizol reagent, further homogenized (TissueTearor; BioSpec Products), and RNA isolated using a Trizol/column method. RNA concentration was quantified (Nanodrop 1000; Thermo Fisher Scientific), converted to cDNA (ABI High‐Capacity cDNA kit), and stored at −20°C until qPCR analysis.

RT‐qPCR was run on the following genes associated with the following: (i) pathways associated with bone formation (*Sost, Col1A1, Runx2, Alp1, Igfr1*), (ii) pathways associated with bone resorption (*Ctsk, OPG, RANKL*), and (iii) osteocyte network/formation (*Cxn43, DMP1, E11*, fimbrin [*PLS1*]). RT‐qPCR was conducted using Taqman primers on a ViiA7 Real‐Time PCR system (Thermo Fisher Scientific). The housekeeping gene (*Ager*) was determined from 18 gene candidates. Selection of this gene was based upon finding no significant change in gene expression for any of the loading treatments conducted. To test for treatment‐induced changes in expression relative to the housekeeping gene, cycle threshold (Ct) values were converted to 2^‐Ct^, and these values compared between the loaded and control limbs within a given load level.

### Statistical analyses

For the μCT and dynamic histomorphometry measures, the effects of loading and load level were tested by a linear mixed model with repeated measures,^(^ ^44^ ^)^ in which the within‐subject factor was limb (loaded and contralateral control) and the between‐subject factor was load group (low, medium, high). Individual paired *t* tests were used to examine the differences between loaded and nonloaded control tibiae for each load level to identify minimum adaptive loads (strains) for cortical and cancellous bone compartments.

For gene expression analyses, all data for each group were first tested for homogeneity in variance using a one‐sample Kolmogorov–Smirnov test. Differences in gene expression between the loaded and control tibiae were analyzed by Wilcoxon signed rank test on the 2^‐ΔCt^ values. Similarly, differences in the ratio for expression of *RANKL* to *OPG* (*RANKL/OPG*) for the loaded *versus* control limbs were tested by Mann–Whitney *U* test using the ratio of the 2^‐ΔCt^ values. Significant differences between limbs were indicated when *p* < 0.05. All analyses were conducted unblinded.

## Results

### Bone formation response was regulated by a load threshold that was greater for the metaphyseal cancellous bone than for the midshaft cortical bone

**Fig 1:**
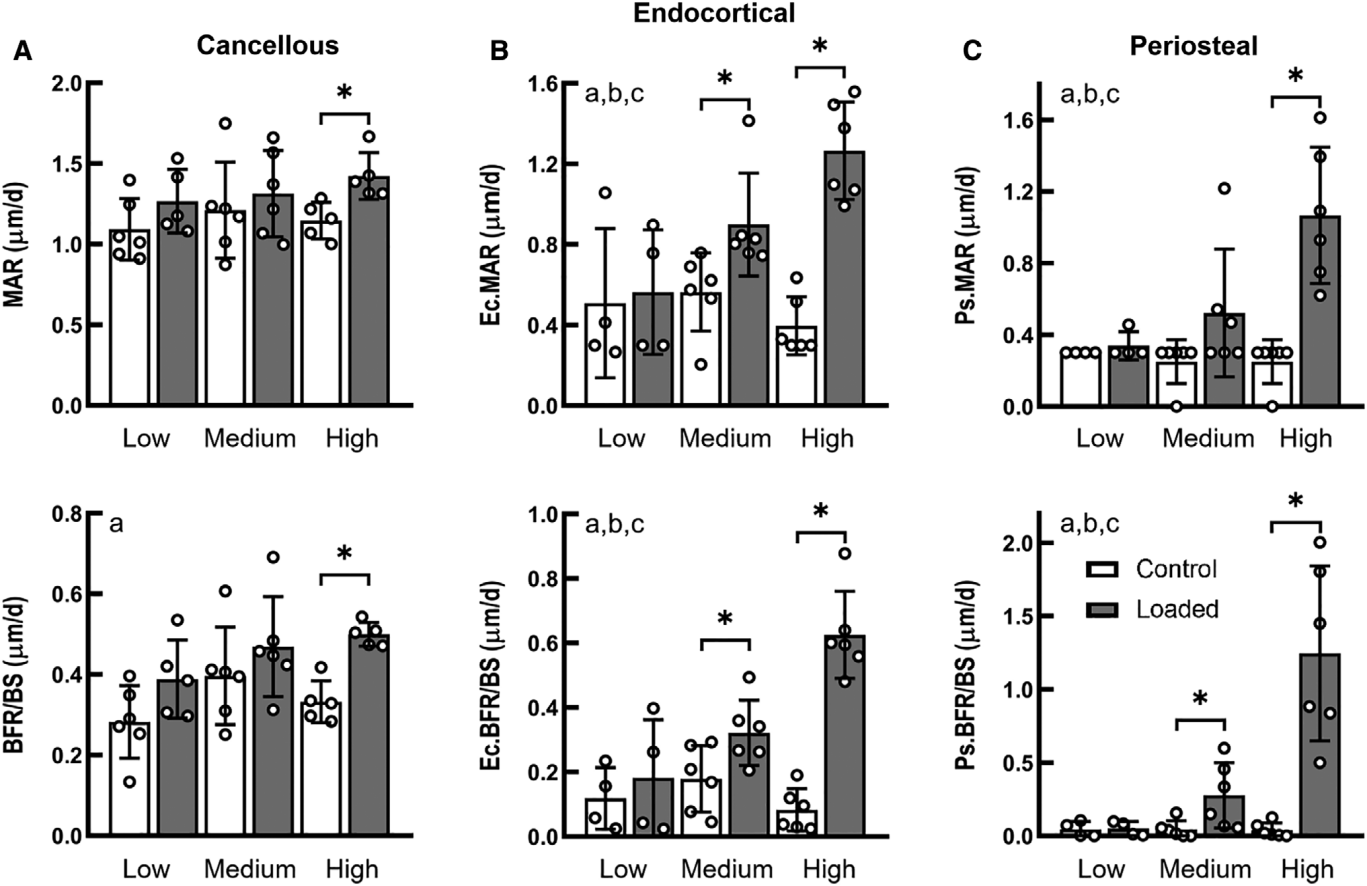
Dynamic histomorphometry showing adaptive changes in mineral apposition rate (MAR) and bone formation rate (BFR/BS) of the proximal cancellous bone tissues (*A*), as well as the endocortical (Ec.) and periosteal (Ps.) bone tissues of the tibial midshaft (*B*,*C*), following applied low (−3.5 N), medium (−5.2 N), and high (−7 N) loads. ^a^Main effect of loading (control vs loaded). ^b^Main effect of load magnitude. ^c^Interaction between loading and load magnitude. *Difference between loaded *versus* control limbs by separate paired *t* tests (*p* < 0.05). Bars: mean ± SD.

For the proximal cancellous bone, bone formation rate (BFR/BS), mineral apposition rate (MAR), and mineralizing surface (MS/BS) increased by 24%, 50%, and 22%, respectively, in only the high load group (Fig. 1 *A*, Supplementary Information Table S1). Load magnitude had a significant effect on the cortical bone formation response to applied mechanical loading (interactive effect of loading and load magnitude, *p* < 0.05; Fig. 1). On the endocortical surface of the tibial midshaft, Ec.BFR/BS and Ec.MAR of the loaded limbs were significantly greater than their contralateral control limbs for the medium‐ and high‐load groups (*p* < 0.05 by paired *t* test), whereas there was no effect of loading on bone formation indices for the low‐load group (Fig. 1*B*). Similarly, on the periosteal surfaces of the tibial midshaft, Ps.BFR/BS and Ps.MS/BS were significantly increased with loading in the medium‐ and high‐load groups but not in the low load‐group (Fig. 1 *C*, Supplementary Information Table S1); Ps.MAR was increased with loading only in the high‐load group (Fig. 1*C*). The increases in the bone formation indices for the high‐load group were significantly greater than for the medium‐ and low‐load groups.

### The metaphyseal cancellous bone had a greater load threshold for bone mass increase relative to the midshaft cortical bone

**Fig 2:**
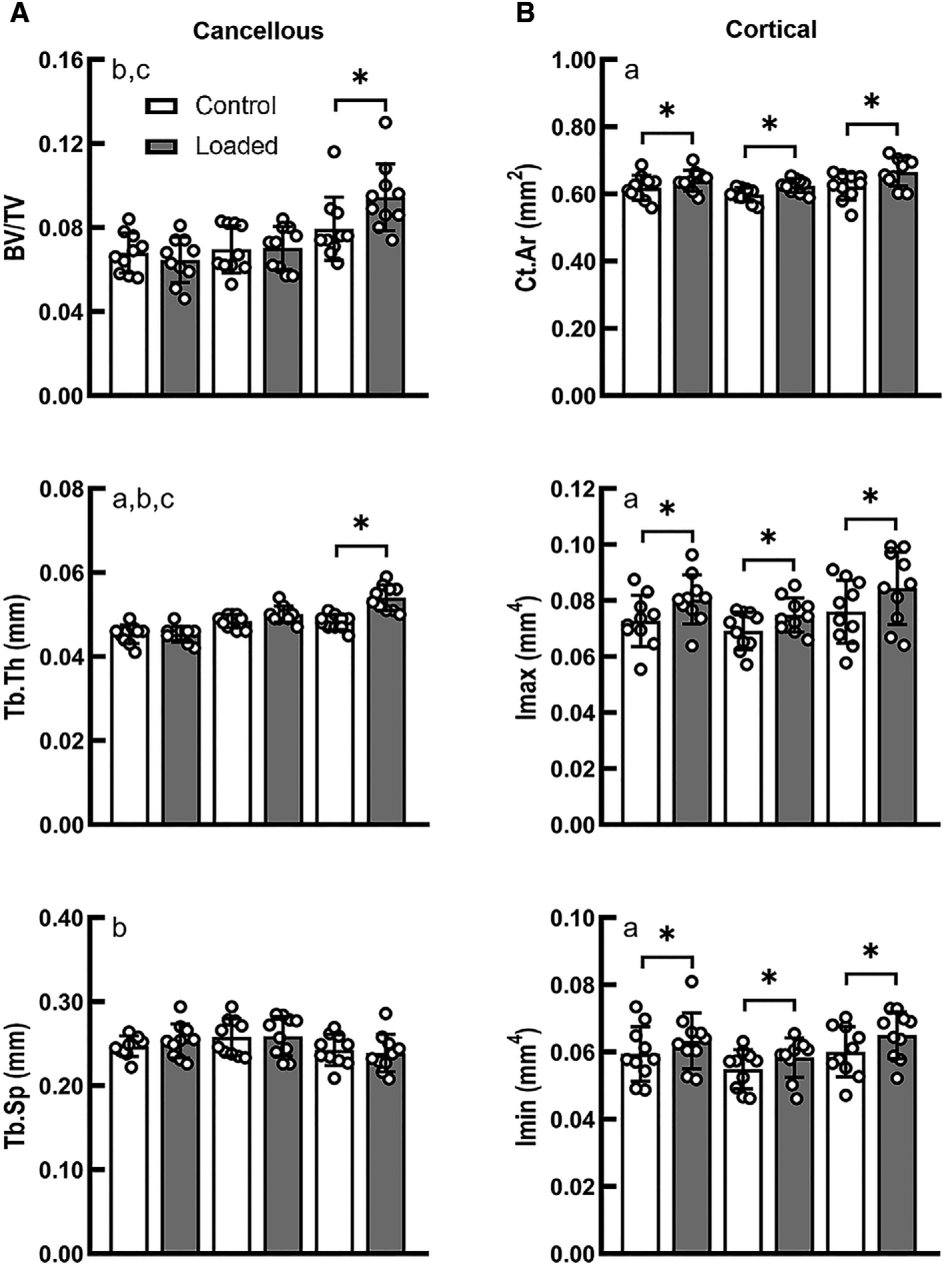
(*A*) μCT analysis showing tibial load‐induced microarchitectural changes of the proximal cancellous bone. BV/TV, bone volume fraction; Tb.Sp, trabecular separation, Tb.Th, trabecular thickness. (*B*) Geometric changes of the midshaft cortical bone. Ct.Ar, cortical area; Imax and Imin, maximum and minimum moments of inertia. ^a^Main effect of loading (control vs loaded). ^b^Main effect of load magnitude. ^c^Interaction between loading and load magnitude. *Difference between loaded *versus* control limbs, by separate paired *t* tests (*p* < 0.05). Bars: mean ± SD.

For the metaphyseal cancellous bone, load magnitude had a significant effect on the changes in BV/TV and Tb.Th in response to applied mechanical loading (interactive effect of loading and load magnitude, *p* < 0.05; Fig. 2). There was no difference in BV/TV between the control limbs of the three load groups. Only under high load did the loaded limb have a greater BV/TV (+18.9%) and Tb.Th (+12.0%) than its contralateral control limb (*p* < 0.05 by paired *t* test; Fig. 2). However, at the midshaft of the tibia, a significant effect of loading was observed for all load groups. Ct.Ar, Tt.Ar, Imax, and Imin were generally increased with applied load for all three load groups (main effect of loading, *p* < 0.05; Fig. 2, Supplementary Information Table S1). The loaded limb had a significantly greater Ct.Ar than its control limb in the low‐ (+3.3%), medium‐, (+4.5%) and high‐ (+7.0%) load groups. Loading led to increases in Imax of 10.6%, 8.2%, and 11.1% for the low‐, medium‐, and high‐load groups, respectively. Loading led to increases in Imin of 6.6%, 6.3%, and 8.2% for the low‐, medium‐, and high‐load groups, respectively.

### Load‐induced alterations in gene expression were different between cortical and cortico‐cancellous tissues dependent upon load magnitude and length of the loading protocol

**Fig 3:**
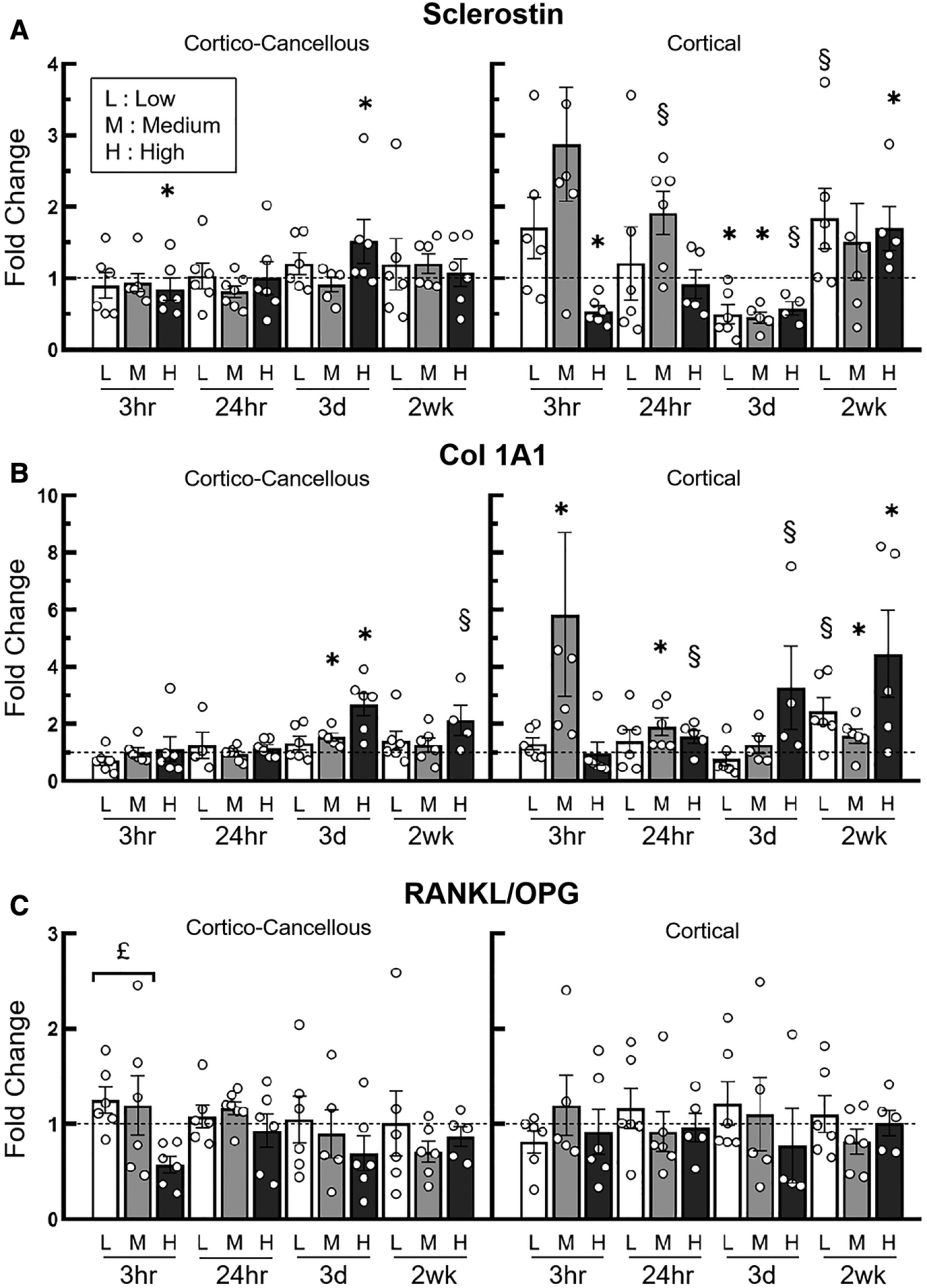
qPCR analysis showing time‐specific fold changes (loaded vs control) in sclerostin expression (*A*), *Col1A1* expression (*B*), and *RANKL/OPG* (*C*) of the proximal metaphyseal cortico‐cancellous and diaphyseal cortical bone tissues of the Tibiae, following loading at low (L), medium (M), and high (H) magnitudes. *p < 0.05, § 0.05 < *p* < 0.1, load *versus* control limbs (Wilcoxon signed ranks test). £: *p* < 0.05 between the groups indicated (*RANKL/OPG*). Bars: mean ± SEM. Note: At the medium load, cortical sclerostin and *Col1A1* data at 3h each had one data point outside of the scale of the y‐axis and was not shown in the figure.

In the metaphyseal cortico‐cancellous tissues, sclerostin expression was only affected by the high load. *Sost* was suppressed at the early time point (3h), showed no change 24h following a single load, and was upregulated at 3 days. By 2wk, *Sost* expression at the high load was unchanged relative to the control limbs. Downregulation or upregulation of *Sost* in the diaphyseal cortical tissues were seen for all load groups (Fig. 3*A*). Specifically, in the diaphyseal cortex, *Sost* expression was reduced 3h following high loads and unchanged in the low‐ and medium‐load groups. Following 3 days of loading, the low‐ and medium‐load groups showed decreased *Sost* expression, whereas the high‐load group showed a statistical trend for this (*p* = 0.07). By 2 weeks, the high‐load group showed increased expression of *Sost* (Fig. 3*A*).

In the cortico‐cancellous tissues load‐induced expression of *Col1A1* was upregulated after 3 days of loading in the medium‐ (1.5‐fold) and high‐load groups (2.7‐fold; Fig. 3*B*). In the cortical diaphyseal tissues, *Col1A1* was upregulated in the medium‐load group 24h after a single load application (2.1‐fold) and following 2 weeks of applied loading (1.6‐fold). Similarly, in the high‐load group trends for increased *Col1A1* were seen in the 24h and 3d groups, but only obtained significant upregulation in the 2wk group (4.4‐fold; Fig. 3*B*). The ratio of *RANKL/OPG* expression was generally unaffected by the application of applied load in both cortico‐cancellous metaphyseal and diaphyseal cortical bone (Fig. 3*C*).

Additional examination of the entire set of genes studied here showed a clear increase in gene expression related to bone formation and establishment of osteocyte networks in the proximal cortico‐cancellous tissues in the 3d group (Supplementary Information Table S2). *DMP1*, *Cxn43*, *Alp1*, and fimbrin were upregulated in both the medium‐load and high‐load groups following 3 days of loading. *Runx2* expression was upregulated in only the high‐load group at 3 days. In the diaphyseal cortical bone volume analyzed, there were no changes in these genes at these same timepoints. However, both *Igfr1*, in addition to *Col1A1* (mentioned above), showed increased expression in the medium‐ and high‐load groups following 2 weeks of loading (Supplementary Information Table S3).

### The adaptive strain thresholds were greater in the metaphyseal cancellous bone than the midshaft cortical bone

**Fig 4:**
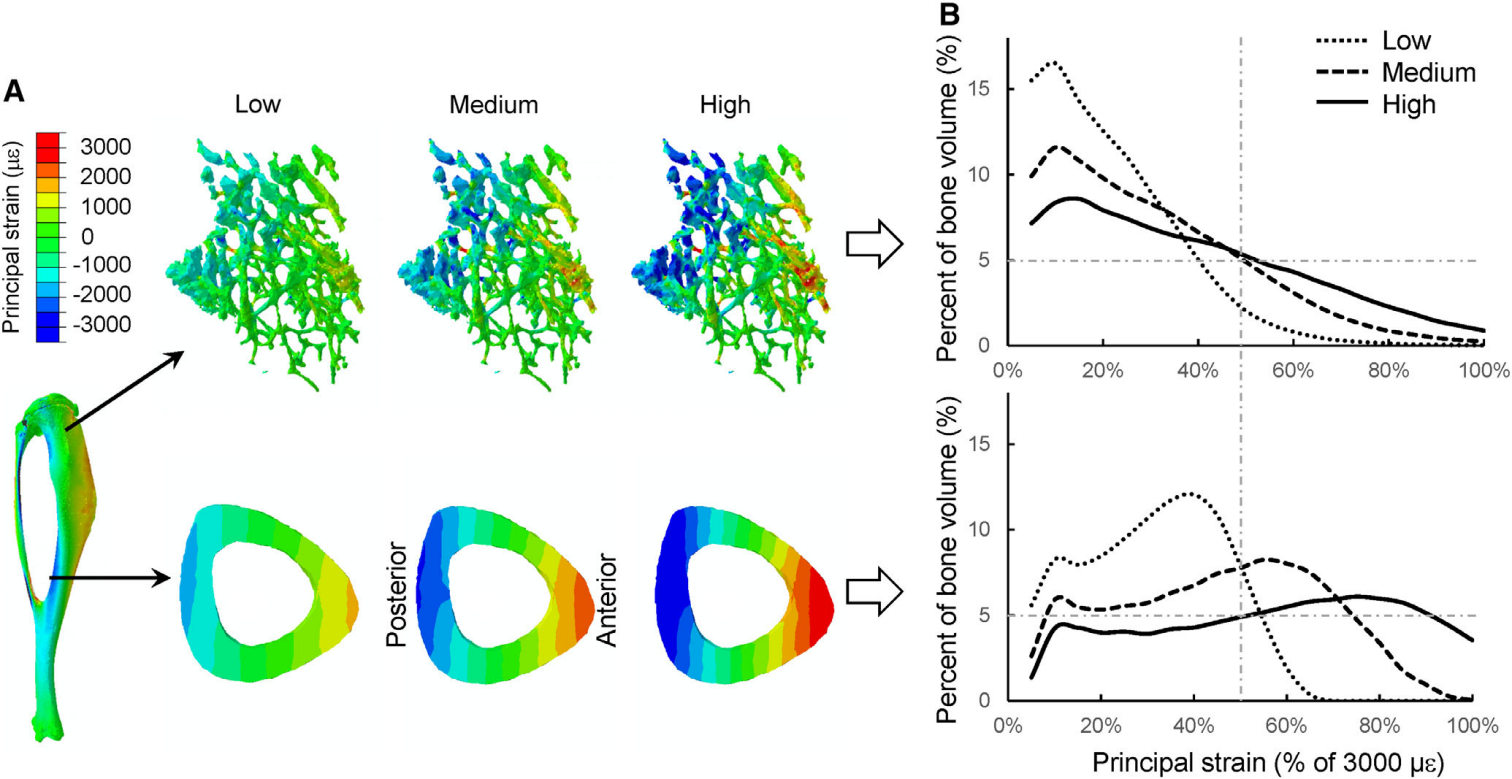
Principal strain distribution (*A*) as well as the frequency distribution of different absolute principal strain magnitudes (*B*) in the proximal metaphyseal cancellous bone and midshaft cortical bone of the Tibiae when subjected to *in vivo* compressive loads of low (−3.5 N), medium (−5.2 N), and high (−7 N) magnitudes. Red and blue indicate tension and compression, respectively. The percentage of bone volume was calculated for every 5% of 3000‐με principal strain. Each curve represents the mean percent of bone volume calculated for seven mice.

FEA showed that the overall strain distribution was different between the proximal metaphyseal cancellous bone and the midshaft cortical bone. Specifically, the fraction of highly strained bone volume was lower in the metaphyseal cancellous bone than the midshaft cortex given the same applied load magnitude (Fig. 4). Under the low, medium, and high loads, there were 4% ± 2%, 16% ± 5% and 30% ± 6% of the metaphyseal cancellous tissues above an absolute principal strain of 1500 με, respectively, whereas the percentages of cortical bone volume exceeding 1500 με were about twofold greater (7% ± 6%, 41% ± 8%, and 60% ± 5%) for the midshaft cortical bone (mean ± SD for seven mice; Fig. 4*B*).

**Fig 5:**
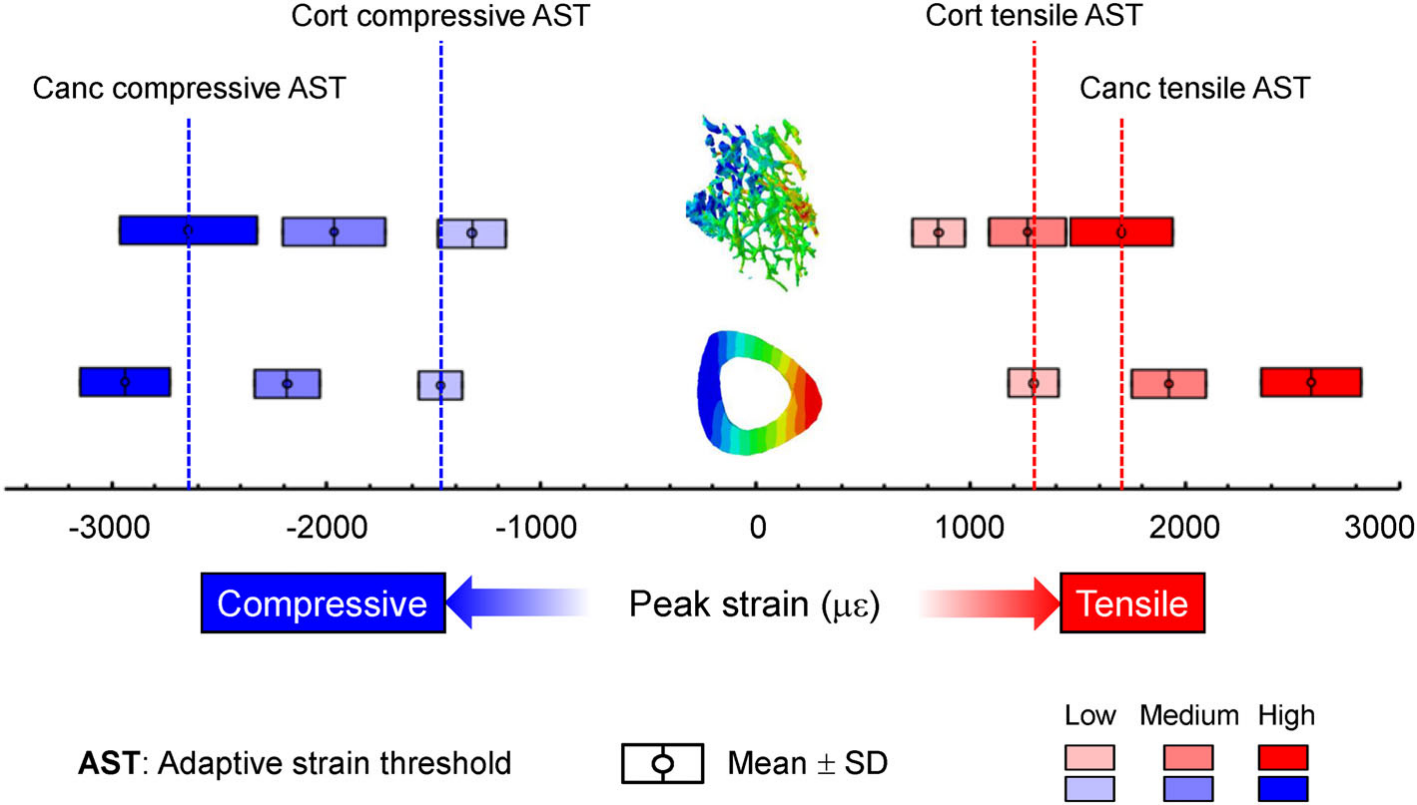
Compressive and tensile adaptive strain thresholds (ASTs) identified for the proximal cancellous bone (Canc) and midshaft cortical bone (Cort) based on their osteogenic load‐related peak compressive or tensile strains (mean values for seven mice).

Load‐induced peak or average strains were lower in the proximal metaphysis than the midshaft for the same applied load magnitude (Table 1). For example, in the midshaft cortical bone, the −3.5 N load generated peak compressive strains of −1469 ± 104 με and peak tensile strains of 1294 ± 117 με, which were significantly greater than those for the proximal cancellous bone (peak compressive: −1322 ± 160 με; peak tensile: 852 ± 120 με) and the proximal cortico‐cancellous bone (peak compressive: −1150 ± 125 με; peak tensile: 948 ± 149 με, *p* < 0.05; Table 1). Comparison of the strains between the metaphysis and midshaft, where load‐induced adaptive responses were first observed, indicate that at the high load the peak strains in the proximal cancellous bone (−2644 ± 319 or 1705 ± 240 με) were significantly greater than the midshaft cortical bone strains at the low load (−1469 ± 104 or 1294 ± 117 με; Fig. 5, Table 1).

## Discussion

This study examined the time‐dependent changes in gene expression, bone formation, and volumetric structural response of cortical and cancellous bone to incremental mechanical loading (low: −3.5 N; medium: −5.2 N; high: −7 N). We sought to determine if the cortical and cancellous bone responses to loading are regulated by different tissue strain thresholds. We also sought to determine whether similar genetic responses were present during cortical and cortico‐cancellous adaptation through load‐induced bone formation. The loading experiment showed that the cortical midshaft bone mass was increased following all load levels examined and bone formation activities were elevated under the medium and high loads. In contrast, for the metaphyseal cancellous bone, only high loads increased bone mass and bone formation indices. Similarly, gene expression associated with inhibition of bone formation (e.g., *Sost*) was altered in the diaphyseal cortical bone by all load levels, but in the metaphyseal cortico‐cancellous bone by only the high load. FEA determined that the tensile and compressive peak or average tissue strains that were osteogenic for the proximal cancellous or cortico‐cancellous bone under the high load were greater than those that were osteogenic for the midshaft cortical tissues under the low load. These results together suggest that the adaptive strain threshold (AST) regulating the structural, cellular, and molecular responses of bone to loading may be greater for the cancellous tissues than for the cortical tissues.

The tibial loading model allows examination of the effects of loading on cortical and cancellous tissues simultaneously within the same bone.^(^ ^26^ ^)^ Similar to several other tibial loading studies,^(^ ^29^, ^31^ ^)^ we find that a greater load magnitude is required to induce an adaptive bone response in the proximal cancellous bone compared with the diaphyseal cortical bone of the tibia. However, the underlying reason for that was not explored previously, particularly from a perspective of tissue strain. Prior *in vivo* tibial loading studies usually performed single‐strain gauge measures to determine a local strain at the medial surface of the tibial midshaft. However, these measures do not reflect the full‐field strain environments in the midshaft cortical bone, let alone strains in the proximal cancellous bone. The present study used a μCT‐based FEA approach, which has been developed and validated previously via *in vivo* strain gauge measurement and comprehensive sensitivity analyses^(^ ^32^, ^39^, ^41^ ^)^ to determine the strain environments within the tibia under different applied load levels. Our results indicate that, under the same applied load magnitude, the amount of highly strained bone tissue is less in the proximal cancellous bone, leading to an overall lower strain level compared with the midshaft cortical bone. Thus, a greater load may be required to increase the overall strain level for the proximal cancellous bone to generate a bone anabolic response. This could explain why the proximal cancellous bone has a greater adaptive load threshold compared with the midshaft cortical bone. Based on the load magnitudes examined in this study, we further identified that the adaptive strain thresholds (e.g., peak tensile or compressive strains) are significantly greater for the proximal cancellous bone than for the midshaft cortical bone (Fig. 5). The adaptive strain threshold values for cortical bone (−1469 με and 1294 με for compressive and tensile peak strains) are in line with previously identified minimum effective strains (~1000με peak strain).^(^ ^16^, ^17^, ^18^ ^)^ These results suggest that cortical and cancellous bone response to mechanical loading may be regulated by different tissue‐level strain thresholds.

The adaptive strain threshold, for either the midshaft cortical or the metaphyseal cancellous VOI, was determined as the peak strain in that VOI where the applied load level induced an adaptive response, whereas lower load levels did not (Fig. 5). Thus, our identified thresholds are likely the “upper bounds” for the exact AST. It would be ideal to test more load levels to narrow down the range for the exact AST, similar to what others have done previously to identify adaptive load thresholds.^(^ ^28^ ^)^ However, because the medium load did not induce an anabolic response in the metaphyseal cancellous bone, the exact AST for the cancellous bone must be greater than the peak strain corresponding to the medium load. Because the medium‐load–induced peak strain in the cancellous bone is greater than or comparable with the low load‐induced peak strain (i.e., the “upper bound” of the exact AST) in the midshaft cortical bone, our conclusion remains the same (Table 1, Fig. 5). Although our data does not allow us to identify an exact AST for cortical or cancellous bone, our general conclusion that cancellous bone may have a greater adaptive strain threshold than cortical bone is still supported.

It is worth noting that the peak strain or the adaptive strain threshold is a local measure, whereas the adaptive response is volumetric. We considered several reasons in rationalizing the use of peak strains to correlate with the volumetric adaptive responses. First, the term adaptive strain threshold used in the current study is similar to the “minimum effective strain” described in the mechanostat theory,^(^ ^15^ ^)^ which was defined according to peak strains. In addition, many studies examining bone adaptation to loading, including those identifying adaptive thresholds of load or strain, commonly report peak strains.^(^ ^16^, ^17^, ^28^ ^)^ The use of peak strain to define adaptive strain threshold makes it feasible to build links between our current findings and the mechanostat theory, as well as with other relevant loading studies. Second, according to beam theory, the peak tensile or compressive strains in a cross‐section of a beam (e.g., tibial midshaft in our case) indicate the overall deformation of the section. Thus, the peak strain, albeit a local strain measure, does reflect the overall mechanical environment of a VOI. Third, there is no definite relation locally between tissue strains and adaptive responses. For example, in the mouse tibial loading model, load‐induced cortical bone formation in the diaphyseal VOI occurs at the endosteal and/or periosteal surfaces where peak cortical strains are located. However, there is no adaptive response in the intracortical bone tissues that experience strain levels between the endosteal and periosteal strain levels. The osteocytes and lacunar‐canalicular networks embedded in the intracortical bone matrix are responsible for sensing matrix deformation (strain) and/or fluid flow to orchestrate bone‐forming osteoblasts and bone‐resorbing osteoclasts located on bone surfaces within a region.^(^ ^6^ ^)^ Thus, the tissue‐level mechanoresponse is a regional rather than a local event. Other models that incorporate fluid flow might be able to shed further light on the relationship between peak strains and the bone anabolic response that we describe here.^(^ ^45^, ^46^ ^)^

The expression of genes related to bone formation and turnover in response to loading is time‐ and load‐threshold–dependent and is different between cortico‐cancellous and cortical tissues. Sclerostin (*Sost*) is a negative regulator of Wnt signaling and subsequently bone formation. It has been shown that mechanical loading can lead to substantial downregulation in sclerostin expression and a decreased number of sclerostin‐positive osteocytes.^(^ ^33^, ^47^, ^48^ ^)^ Holguin and colleagues examined time‐specific expression of sclerostin in the tibial diaphysis in response to compressive loading and found in 5‐month‐old female C57BL/6 mice that sclerostin was downregulated at 4 and 24h after a single bout of loading and downregulated 3 and 5 days after repeated daily loading bouts.^(^ ^36^ ^)^ Similarly, we observed downregulation of sclerostin in diaphyseal cortical bone 3h and 3 days following a single‐load or multiple loading bouts (Fig. 3*A*). Consistent with the observed structural responses, sclerostin downregulation was observed here in the diaphyseal cortical bone for the low, medium, and high loads, whereas sclerostin downregulation in the proximal cortico‐cancellous bone only occurred in the high load. The less sensitive changes in sclerostin expression to incremental loading in the proximal cortico‐cancellous tissues relative to the diaphyseal cortical tissues suggest that sclerostin expression in cancellous and cortical tissues may also be regulated by different strain thresholds. Similarly, sensitivity to load‐induced strains in relation to immunohistochemical sclerostin expression was observed in tibial and ulnar cortical bone, where the heterogeneous regional differences in sclerostin expression mirrored the regional differences between high and low cortical bone strains.^(^ ^33^, ^47^ ^)^ We also observed load‐induced upregulation of sclerostin 2 weeks following loading, which seems unexpected, but could be explained by reduced strain levels as bone adaptation saturates with applied loading.^(^ ^39^ ^)^ *Col1A1*, a primary bone matrix gene that characterizes matrix synthesis, was upregulated at earlier time points (24h) by the medium load in the diaphyseal cortical bone than in the proximal cortico‐cancellous tissues (3 days), suggesting that bone formation response to loading initiates at lower strains more quickly in diaphyseal cortical compared with metaphyseal cancellous bone. The upregulation of *Col1A1* after 2 weeks of loading in diaphyseal cortical bone is similar to what others have observed.^(^ ^35^, ^49^ ^)^ *RANKL/OPG* was not generally altered by applied loads of varied magnitudes except it was reduced in cortico‐cancellous tissues 3h following high load relative to low load. Unchanged expression of *RANKL/OPG* following loading was also observed in other studies^(^ ^49^ ^)^ and suggests that applied loading primarily impacts bone formation because bone resorption markers (*RANKL*, *OPG*, *Ctsk*) were not consistently affected by applied loading. In osteocytes, the activation of *Piezo1* leads to increased *Wnt1* expression, which in turn activates the Wnt signaling pathway stimulating new bone formation.^(^ ^50^ ^)^ It would be of interest to examine *Piezo1* and *Wnt1* expression in response to varied load levels in future studies.

Our study has a few limitations. First, our structural analysis in the metaphysis focused on cancellous bone, whereas our gene‐level analysis examined cortico‐cancellous bone because of the difficulty of isolating mouse tibial cancellous bone for qPCR analysis. Therefore, the structural and gene analyses may not fully reflect each other because the cortico‐cancellous gene analysis considers the cortical, cancellous, and marrow cell strain environments. The average strains modeled for the cortico‐cancellous and cancellous VOIs are actually quite similar (Table 1). Absolute peak compressive strains are lower and peak tensile strains are greater in the cortico‐cancellous tissues compared with cancellous bone volumes. Tissue strain differences between the proximal cancellous and cortico‐cancellous strains in each load group are less than the differences between load groups. Furthermore, the changes in *Sost* and *Col1A1* in the cortico‐cancellous bone are reflective of the high strain threshold for the volumetric changes in bone mass. This part of the study was conducted prior to subsequent publications presenting methods for isolating mouse tibial cancellous bone for gene expression analyses.^(^ ^51^ ^)^

Second, the adaptive strain thresholds identified for cortical or cancellous bone in the current study may not be taken as universal values because many mechanical stimulation parameters of the applied loading protocol (e.g., frequency, rate, duration, and rest insertion) can affect the extent of the bone's response.^(^ ^26^ ^)^ For example, insertion of a rest interval appeared to lower the “strain threshold” at which bone formation could be initiated.^(^ ^9^, ^52^ ^)^ However, our main findings should not be affected because the cortical and cancellous tissues in the tibial loading model receive the exact same form of externally applied mechanical signals. Also, our recent study found that insertion of a short rest between load cycles does not affect cortical and cancellous bone adaptation in this tibial loading model.^(^ ^44^ ^)^

In summary, our results, based on the tibial loading model, suggest that the adaptive strain thresholds may be greater for cancellous than for cortical bone. Future studies using multiscale bone mechanical models may be required to confirm this by examining cortical and cancellous tissue strains in relation to cell‐level stimuli to determine why greater tissue strains are required to elicit a load‐induced volumetric increase in cancellous relative to cortical bone in the mouse axial tibial loading model.

## AUTHOR CONTRIBUTIONS

**Haisheng Yang:** Conceptualization; data curation; formal analysis; investigation; methodology; project administration; validation; writing‐original draft; writing‐review & editing. **Whitney Bullock:** Conceptualization; data curation; formal analysis; investigation; methodology; project administration; supervision; writing‐original draft. **Alexandra Myhal:** Formal analysis; investigation; methodology; visualization; writing‐review & editing. **Philip DeShield:** Conceptualization; investigation; methodology; writing‐review & editing. **Daniel Duffy:** Conceptualization; formal analysis; investigation; methodology; writing‐review & editing. **Russell Main:** Conceptualization; data curation; formal analysis; funding acquisition; investigation; methodology; project administration; resources; software; supervision; validation; writing‐original draft; writing‐review & editing.

## Conflict of Interest

The authors declare that there is no conflict of interests that could be perceived as prejudicing the impartiality of the research reported.

### PEER REVIEW

The peer review history for this article is available at https://publons.com/publon/10.1002/jbm4.10489.

## Supporting information


**Figure S1.** Experimental design and analyses for the mice included in this study. *In vivo* dynamic compressive loads at Low (−3.5 N), Medium (−5.2 N) and High levels (−7 N) were applied by the actuator at the foot of the left hindlimb and transmitted through the tibia to the distal femur (*A* and *B*). A single load session consisted of 216 total load events (4 cycles at 4 Hz followed by a 5 second rest phase at ‐1 N, repeated 54 times) (*B*). Mice were subjected to dynamic cyclic loading for a single loading session (3 h, 24 h), three loading sessions over 3 days (3d), or 10 load sessions over 2 weeks (5d/week) (*C*). Load‐induced changes in the proximal metaphyseal cancellous bone (canc) and midshaft cortical bone (cort) following loading were examined by microCT and histomorphometry (*D*). The diaphysis (cort) and metaphysis (cortico‐canc) of the loaded and control tibiae were analyzed for changes in the expression of genes related to bone formation and resorption (*D*).

**Table S1.** Additional microCT results (Tt.Ar, Ma.Ar, Ct.Th) for midshaft cortical geometry and histomorphometry parameters (MS/BS) for the midshaft cortical and proximal metaphyseal cancellous bone of the mouse tibiae under axial compressive loads of −3.5 N, −5.2 N and − 7 N.

**Table S2.** qPCR analysis showing time‐specific fold changes (loaded vs control) in gene expression of the metaphyseal cortico‐cancellous tissues of the tibiae, following loading at Low (L), Medium (M) and High (H) magnitudes. Bold: *p* < 0.05, load vs. control limbs (Wilcoxon Signed Ranks Test).

**Table S3.** qPCR analysis showing time‐specific fold changes (loaded vs control) in gene expression of the diaphyseal cortical tissues of the tibiae, following loading at Low (L), Medium (M) and High (H) magnitudes. Bold: *p* < 0.05, load vs. control limbs (Wilcoxon Signed Ranks Test).
