# Supplementary figures and images for: Cancellous Bone May Have a Greater Adaptive Strain Threshold Than Cortical Bone
Source: JBMR Plus. 2021 Mar 30;5(5):e10489. doi: 10.1002/jbm4.10489 (PMC8101616; doi:10.1002/jbm4.10489)

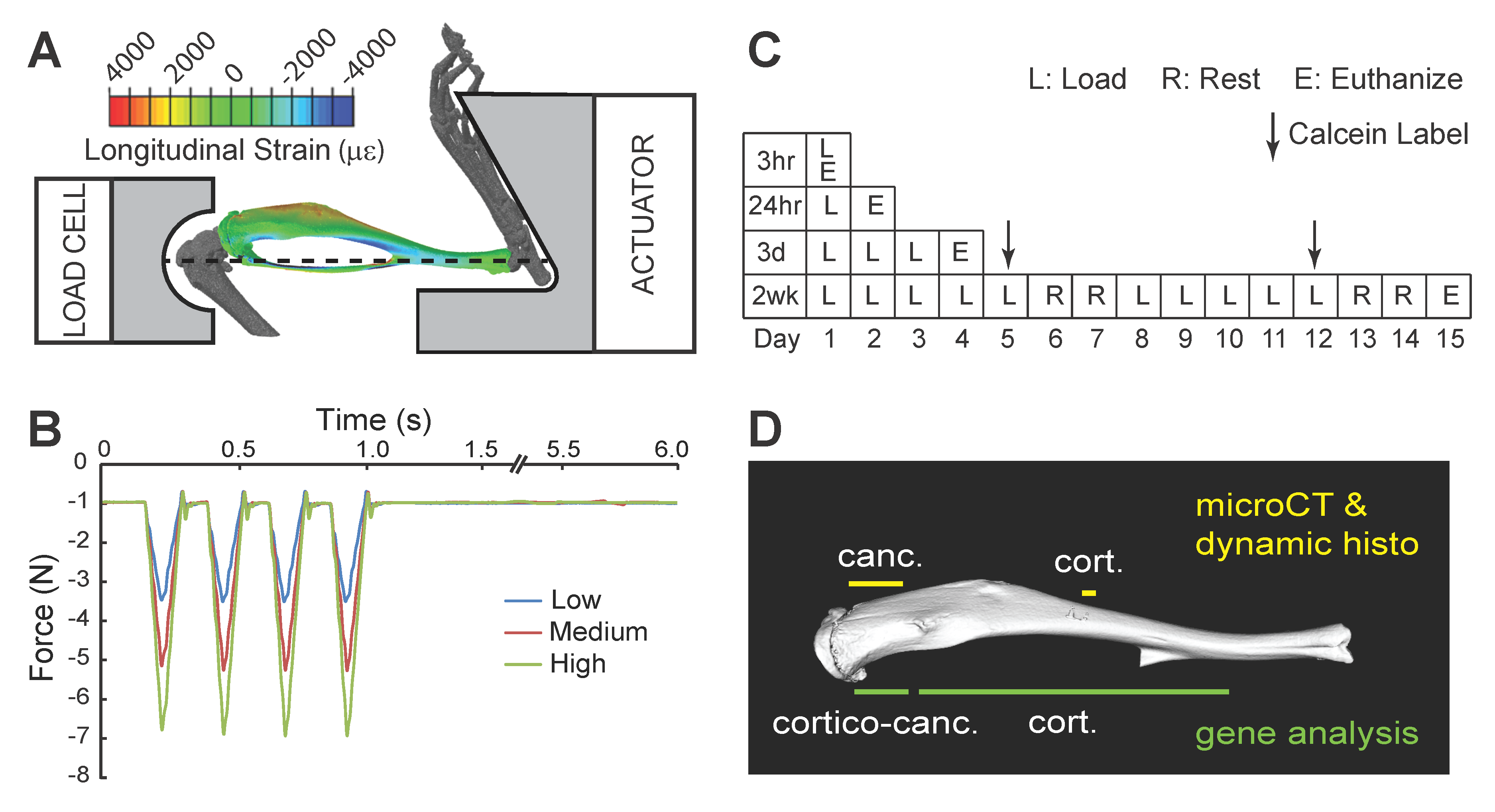

Supplement: Supplementary file 1 — Figure S1. Experimental design and analyses for the mice included in this study. In vivo dynamic compressive loads at Low (−3.5 N), Medium (−5.2 N) and High levels (−7 N) were applied by the actuator at the foot of the left hindlimb and transmitted through the tibia to the distal femur (A and B). A single load session consisted of 216 total load events (4 cycles at 4 Hz followed by a 5 second rest phase at ‐1 N, repeated 54 times) (B). Mice were subjected to dynamic cyclic loading for a single loading session (3 h, 24 h), three loading sessions over 3 days (3d), or 10 load sessions over 2 weeks (5d/week) (C). Load‐induced changes in the proximal metaphyseal cancellous bone (canc) and midshaft cortical bone (cort) following loading were examined by microCT and histomorphometry (D). The diaphysis (cort) and metaphysis (cortico‐canc) of the loaded and control tibiae were analyzed for changes in the expression of genes related to bone formation and resorption (D). [file JBM4-5-e10489-s004.tif]
