# Supplementary material for: Cancellous Bone May Have a Greater Adaptive Strain Threshold Than Cortical Bone
Source: JBMR Plus. 2021 Mar 30;5(5):e10489. doi: 10.1002/jbm4.10489 (PMC8101616; doi:10.1002/jbm4.10489)
Supplement: Supplementary file 2 — Table S1. Additional microCT results (Tt.Ar, Ma.Ar, Ct.Th) for midshaft cortical geometry and histomorphometry parameters (MS/BS) for the midshaft cortical and proximal metaphyseal cancellous bone of the mouse tibiae under axial compressive loads of −3.5 N, −5.2 N and − 7 N. [file JBM4-5-e10489-s001.docx]

**Table S1** Additional microCT results (Tt.Ar, Ma.Ar, Ct.Th) for midshaft cortical geometry and histomorphometry parameters (MS/BS) for the midshaft cortical and proximal metaphyseal cancellous bone of the mouse tibiae under axial compressive loads of -3.5 N, -5.2 N and -7 N.

| Parameters | Low (-3.5 N) | |  | Medium (-5.2 N) | |  | High (-7 N) | |
| --- | --- | --- | --- | --- | --- | --- | --- | --- |
|  | Control | Loaded |  | Control | Loaded |  | Control | Loaded |
| Tb.MS/BS | 0.257±0.067 | 0.286±0.061 |  | 0.324±0.022 | 0.356±0.045 |  | 0.290±0.040 | 0.354±0.032^*^ |
| Ec.MS/BS | 0.226±0.121 | 0.253±0.171 |  | 0.302±0.117 | 0.359±0.069 |  | 0.192±0.115 | 0.496±0.059^*^ |
| Ps.MS/BS | 0.152±0.172 | 0.142±0.143 |  | 0.148±0.197 | 0.492±0.260^*^ |  | 0.128±0.167 | 1.124±0.218^*^ |
| Tt.Ar | 0.941±0.061 | 0.981±0.060^*^ |  | 0.915±0.051 | 0.944±0.045^*^ |  | 0.957±0.065 | 0.992±0.064^*^ |
| Ma.Ar | 0.322±0.029 | 0.341±0.032^*^ |  | 0.317±0.036 | 0.319±0.030 |  | 0.335±0.032 | 0.326±0.028 |
| Ct.Th | 0.220±0.012 | 0.228±0.007 |  | 0.213±0.014 | 0.224±0.013^*^ |  | 0.221±0.011 | 0.232±0.010^*^ |

Data are presented as mean±SD values.

MS/BS: mineralizing surface. Tb: proximal metaphyseal cancellous bone. Ec: midshaft endocortical. Ps: midshaft periosteal.

Tt.Ar: midshaft total area (mm^2^). Ma.Ar: midshaft medullary area (mm^2^). Ct.Th: midshaft cortical thickness (mm).

^*^ p < 0.05 vs. respective control, by paired t-test.
