# Supplementary material for: Cancellous Bone May Have a Greater Adaptive Strain Threshold Than Cortical Bone
Source: JBMR Plus. 2021 Mar 30;5(5):e10489. doi: 10.1002/jbm4.10489 (PMC8101616; doi:10.1002/jbm4.10489)
Supplement: Supplementary file 3 — Table S2. qPCR analysis showing time‐specific fold changes (loaded vs control) in gene expression of the metaphyseal cortico‐cancellous tissues of the tibiae, following loading at Low (L), Medium (M) and High (H) magnitudes. Bold: p < 0.05, load vs. control limbs (Wilcoxon Signed Ranks Test). [file JBM4-5-e10489-s002.docx]

| Gene | Loading | | | | | | | | | | | | | | |
| --- | --- | --- | --- | --- | --- | --- | --- | --- | --- | --- | --- | --- | --- | --- | --- |
|  | 3hr | | |  | 24hr | | |  | 3d | | |  | 2wk | | |
|  | L | M | H |  | L | M | H |  | L | M | H |  | L | M | H |
| Ctsk | 0.8 | **1.5** | 1.5 | 1.2 | | **0.6** | 1.1 | 1.2 | | 1.1 | 1.4 | 1.3 | | 1.2 | 1.8 |
| Cxn43 | 0.8 | 1.1 | 1.1 | 1.2 | | 0.8 | 1.0 | 1.1 | | **1.2** | **2.4** | 1.2 | | 1.0 | 1.5 |
| DMP1 | 0.9 | 1.8 | 2.2 | 1.2 | | 0.7 | 0.9 | 1.4 | | **1.2** | **1.6** | 1.5 | | 1.4 | 1.9 |
| E11 | 0.8 | 0.7 | 1.1 | 1.3 | | **0.8** | 1.4 | 0.9 | | 1.3 | 1.3 | 1.2 | | 0.9 | 0.8 |
| Fimbrin | **0.6** | 1.2 | 1.0 | 1.1 | | 0.8 | 0.9 | 1.3 | | **1.6** | **5.2** | 1.4 | | 1.1 | **2.1** |
| Igfr1 | 0.6 | 0.7 | **0.7** | 1.1 | | 0.9 | 0.7 | 1.0 | | 1.3 | 1.8 | 1.1 | | 0.9 | 1.0 |
| OPG | 0.6 | 1.0 | 3.7 | 1.1 | | 0.6 | 1.3 | 1.5 | | 1.7 | **4.1** | **1.7** | | **1.6** | 1.9 |
| Col1A1 | 0.7 | 1.0 | 1.1 | 1.2 | | 1.0 | 1.1 | 1.3 | | **1.5** | **2.7** | 1.4 | | 1.3 | 2.1 |
| RANKL | 0.7 | 1.2 | 1.5 | 1.2 | | 0.7 | 0.9 | 1.2 | | 1.2 | 2.0 | 1.4 | | 1.1 | **1.8** |
| Runx2 | 0.8 | 1.1 | 0.9 | 1.2 | | 0.9 | 0.9 | 1.4 | | 1.3 | **2.1** | 1.3 | | 1.1 | 1.3 |
| Sost | 0.9 | 1.0 | **0.8** | 1.0 | | 0.8 | 1.0 | 1.2 | | 0.9 | **1.5** | 1.2 | | 1.2 | 1.1 |
| Alp1 | 0.7 | 1.1 | 1.2 | 0.9 | | 0.9 | 1.2 | 1.4 | | **1.6** | **2.1** | 1.3 | | 1.1 | 1.7 |
| RANKL/OPG | 1.3 | 1.2 | 0.6 | 1.1 | | 1.2 | 0.9 | 1.0 | | 0.9 | 0.7 | 1.0 | | 0.7 | 0.9 |

**Table S2:** qPCR analysis showing time-specific fold changes (loaded vs control) in gene expression of the metaphyseal cortico-cancellous tissues of the tibiae, following loading at Low (L), Medium (M) and High (H) magnitudes. Bold: p<0.05, load vs. control limbs (Wilcoxon Signed Ranks Test).
