# Supplementary material for: Cancellous Bone May Have a Greater Adaptive Strain Threshold Than Cortical Bone
Source: JBMR Plus. 2021 Mar 30;5(5):e10489. doi: 10.1002/jbm4.10489 (PMC8101616; doi:10.1002/jbm4.10489)
Supplement: Supplementary file 4 — Table S3. qPCR analysis showing time‐specific fold changes (loaded vs control) in gene expression of the diaphyseal cortical tissues of the tibiae, following loading at Low (L), Medium (M) and High (H) magnitudes. Bold: p < 0.05, load vs. control limbs (Wilcoxon Signed Ranks Test). [file JBM4-5-e10489-s003.docx]

**Table S3:** qPCR analysis showing time-specific fold changes (loaded vs control) in gene expression of the diaphyseal cortical tissues of the tibiae, following loading at Low (L), Medium (M) and High (H) magnitudes. Bold: p<0.05, load vs. control limbs (Wilcoxon Signed Ranks Test).

| Gene | Loading | | | | | | | | | | | | | | |
| --- | --- | --- | --- | --- | --- | --- | --- | --- | --- | --- | --- | --- | --- | --- | --- |
|  | 3hr | | |  | 24hr | | |  | 3d | | |  | 2wk | | |
|  | L | M | H |  | L | M | H |  | L | M | H |  | L | M | H |
| Ctsk | 1.3 | **5.1** | 1.2 | 1.3 | | **1.9** | 0.8 | 0.9 | | 0.6 | 0.6 | 2.1 | | 1.1 | **2.4** |
| Cxn43 | 1.2 | 2.9 | 0.9 | 1.3 | | **2.2** | 1.1 | 0.7 | | 0.9 | 1.4 | 2.1 | | **1.5** | 3.4 |
| DMP1 | 1.6 | **5.8** | 1.1 | 0.9 | | **2.3** | 0.9 | 0.8 | | 0.6 | 1.1 | 2.2 | | 1.7 | 2.1 |
| E11 | 0.8 | 0.9 | 1.3 | 4.8 | | 1.1 | 0.9 | 1.7 | | **1.7** | 1.7 | 1.3 | | **2.1** | 1.1 |
| Fimbrin | 1.3 | **7.7** | 1.1 | 1.4 | | **2.6** | 1.3 | 1.0 | | 1.0 | 2.0 | 2.3 | | 1.4 | 2.7 |
| Igfr1 | 1.0 | 2.6 | 0.9 | 1.2 | | 1.2 | 1.0 | 0.8 | | 1.2 | 1.3 | 1.8 | | **2.2** | **2.2** |
| OPG | **1.5** | 4.7 | 1.0 | 1.3 | | **2.6** | 1.1 | 0.9 | | 1.0 | 1.5 | 2.2 | | **1.6** | 2.4 |
| Col1A1 | 1.3 | **5.8** | 1.0 | 1.4 | | **1.9** | 1.6 | 0.8 | | 1.3 | 3.3 | 2.4 | | **1.6** | **4.4** |
| RANKL | 1.1 | **4.8** | 0.9 | 1.2 | | **2.1** | 1.0 | 0.9 | | 0.9 | 0.8 | 2.2 | | 1.3 | 2.3 |
| Runx2 | 1.6 | **5.1** | 1.1 | 1.2 | | **2.1** | 1.0 | 0.8 | | 1.1 | 1.1 | 2.5 | | 1.4 | **2.6** |
| Sost | 1.7 | 2.9 | **0.5** | 1.2 | | 1.9 | 0.9 | **0.5** | | **0.5** | 0.6 | 1.8 | | 1.5 | **1.7** |
| Alp1 | 1.5 | **4.6** | 0.9 | 1.3 | | **2.5** | 1.6 | 0.8 | | 1.1 | 1.7 | 2.5 | | 1.5 | 3.6 |
| RANKL/OPG | 0.8 | 1.2 | 0.9 | 1.2 | | 0.9 | 1.0 | 1.2 | | 1.1 | 0.8 | 1.1 | | 0.8 | 1.0 |

Note: The results for the Medium group at 3hr and 24hr were deemed to be anomalous given the extreme changes in gene expression across many genes without similar findings for the High load group, which we would expect to show similar if not greater changes in the expression of similar genes. The data for these groups are presented here, regardless, but we do not discuss them in the manuscript.
